# Response to Nivolumab followed by complete cytoreductive surgery with HIPEC resulted in long-term survival in a patient with sarcomatoid-predominant biphasic peritoneal mesothelioma. A case report

**DOI:** 10.1016/j.ijscr.2023.108359

**Published:** 2023-05-27

**Authors:** Paul H. Sugarbaker

**Affiliations:** Washington Cancer Institute, Washington, DC, USA

**Keywords:** Peritoneal surface malignancy, Peritoneal mesothelioma, Sarcomatoid mesothelioma, Biphasic mesothelioma, Heterogeneous response, Case report

## Abstract

**Introduction and importance:**

Sarcomatoid-predominant biphasic peritoneal metastases is a rapidly progressing and deeply invasive variant of this disease with survival measured in months. Although cytoreductive surgery (CRS) and hyperthermic intraperitoneal chemotherapy (HIPEC) is a standard of care for epithelioid peritoneal mesothelioma, the sarcomatoid variant is so aggressive, the standard treatment is not recommended. Immunotherapy has recently been utilized for pleural mesothelioma. Partial responses to immunotherapy may be combined with CRS to achieve a favorable outcome in sarcomatoid-predominant peritoneal mesothelioma.

**Case presentation:**

A 39-year-old woman developed an expanding abdomen. A 10 cm pelvic mass was removed by hysterectomy. With an initial diagnosis of advanced ovarian cancer, she was treated with cisplatin plus paclitaxel. Disease progression led to a review of her original pathology and a repeat biopsy which showed biphasic peritoneal mesothelioma with sarcomatoid predominance. Treatment with Nivolumab was transiently beneficial. Repeat CT 8 months later showed partial bowel obstruction and necrotic expanding tumor masses that were partially calcified. CRS with HIPEC and normothermic long-term intraperitoneal pemetrexed (NIPEC) plus intravenous cisplatin resulted in a 5-year disease-free survival.

**Clinical discussion:**

The specimens removed at CRS showed marked progression within large masses. Smaller masses resected with CRS showed fibrosis and calcification. The response to Nivolumab was heterogeneous with smaller masses with good blood supply adequately treated but larger masses markedly progressed.

**Conclusions:**

A combination of partial response to immunotherapy with a complete CRS plus HIPEC and NIPEC can result in a long-term favorable outcome.

## Highlights

• Epithelioid peritoneal mesothelioma is treated by CRS, HIPEC and NIPEC.
• Peritoneal mesothelioma with a biphasic pathology has few treatment options.
• Monoclonal antibodies used as PD-1 antagonists can activate an immune response.
• Partial response with Nivolumab combined with complete CRS showed benefit.

## 1 Introduction

In the management of peritoneal metastases, the low-grade malignancies such as cystic mesothelioma and pseudomyxoma peritonei are adequately treated with complete CRS and perioperative intraperitoneal chemotherapy. In contrast, high-grade cancer with peritoneal metastases had the most favorable outcome when the systemic chemotherapy is maximally effective and the cytoreductive surgery is complete [1]. To date, success in combining targeted monoclonal antibody treatments with cytoreductive surgery in patients with peritoneal surface malignancy has not as yet been reported. In this case I report a heterogeneous response to Nivolumab combined with complete cytoreductive surgery followed by hyperthermic intraperitoneal chemotherapy (HIPEC) and normothermic intraperitoneal chemotherapy (NIPEC) to result in a five-year survival in a patient with high-grade sarcomatoid-predominant biphasic peritoneal mesothelioma [2,3]. The implications of this combined treatment for further improvements in the management of malignant mesothelioma are presented.

## 2 Materials and methods

Data on this patient was prospectively recorded and then retrospectively reviewed at an academic institution. This research work has been reported in line with the SCARE 2020 criteria [4]. This study was registered as a case report on the www.researchregistry.com website with UIN researchregistry8983.

## 3 Case presentation

Autumn 2017. This 39-year-old otherwise healthy woman began to note abdominal bloating and occasional vaginal bleeding. This occurred soon after the placement of an intrauterine device. On 12/1/2017, she went back to her gynecologist to check for placement of the intrauterine device. Although the IUD placement was satisfactory a pelvic mass was noted and the patient was sent immediately for an abdominal and pelvic CT scan. The patient was told that she had a grapefruit-size mass within the pelvis.

December 15, 2017. The patient was taken to the operating room with a preoperative diagnosis of ovarian cancer. A hysterectomy and bilateral salpingo-oophorectomy were performed. The patient was told that 99 % of the tumor had been removed. The pathology report was interpreted as high-grade ovarian malignancy.

December 26, 2017. Two weeks later in the postoperative period, the patient developed a fever to 103.7 °F, vomiting, and shortness of breath. The patient was taken back to the operating room for evacuation of purulent material from her pelvis. She was released from the hospital after one week.

January 11. 2018 and February 1, 2018. The patient was treated with two cycles of systemic chemotherapy with a diagnosis of ovarian cancer with paclitaxel and carboplatin. The chemotherapy treatments were poorly tolerated and resulted in a 24-pound weight loss.

February 12, 2018. CT scan was performed which showed recurrence of tumors in the pelvis.

February 27, 2018. PET-CT was performed that showed progression of the disease within the pelvis. A decision was made that the systemic chemotherapy for presumed ovarian malignancy was ineffective.

February 28, 2018. Referral to MD Anderson Cancer Center brought about a review of the pathology slides from the original surgery. Also, a repeat CT-guided percutaneous biopsy was performed. The revised diagnosis was biphasic malignant peritoneal mesothelioma involving ovaries, para-ovarian tissue, and uterine serosa. The final immunostains were positive for WT-1, D2–40, thrombomodulin, CK5/6, B72.3, and calretinin. MOC-31, BER-EP4, CD15, CEA, PAX-8, GATA3, and ER.BAP1 showed absence of staining. FISH studies showed homozygous deletion of P16. Final diagnosis was biphasic mesothelioma with cartilaginous metaplasia. There was a predominant sarcomatoid histology.

March 15, 2018. The patient was started on Nivolumab 240 mg intravenously every 2 weeks [5]. The molecular treatments were continued until October 17, 2018. Initially, there was a response with partial shrinkage of abdominal and pelvic tumor masses. Tumor expression of PD-1 or PD-L1 within the tumor was undetermined.

**Fig. 1:**
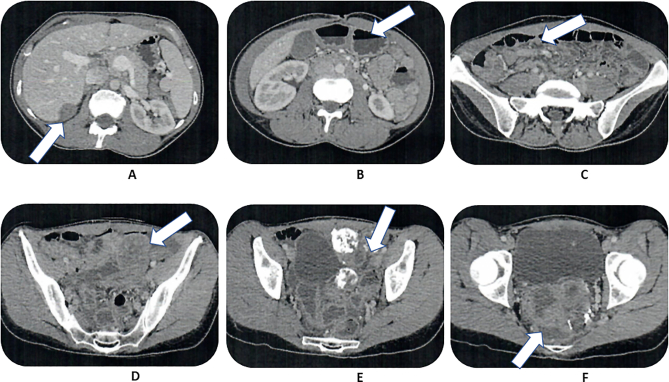
a. Mass right subhepatic space. b. Partial large bowel obstruction, no hydronephrosis. c. Small nodules associated with small bowel. d. Left lower quadrant mass associated with descending colon. e. Heavily calcified masses filling the left hemipelvis. f. Partially calcified mass filling the cul-de-sac and obstructing the rectum.

October 29, 2018. A repeat CT scan was performed and multiple cuts are reproduced as Fig. 1. Filling defects in the right retrohepatic space, nodules associated with the small bowel and its mesentery, diffuse calcification of nodules especially within the pelvis and obstruction of the rectosigmoid colon by masses of mesothelioma was demonstrated by the CT.

November 5, 2018. The patient noted difficulty in passing stool. She required morphine for pain. Large bowel obstruction was partially relieved by coffee enemas performed on a daily basis.

November 12, 2018. Tumor markers CEA = 1.0 μg/ml, CA125 = 20.4 units/ml, CA19–9 = 14.0 units/ml, and CA15–3 = 16.3 units/ml.

November 13, 2018. The patient underwent a 12-hour cytoreductive surgical procedure with bidirectional HIPEC and the placement of a permanent intraperitoneal port for NIPEC long-term [2,3,6]. Ureteral stents were placed at the initiation of the surgery. A complete stripping of the pelvic peritoneum, descending colon, rectosigmoid colon, and rectal resection was necessary. A resection of the apex of the vagina was required to remove disease infiltrating this structure. A cholecystectomy, appendectomy, greater omentectomy, wedge resection of a liver mass from the surface of segment 7 of the liver, small bowel resection with anastomosis, and suture repair of the bladder were performed. The suture repair of the bladder was required because the prior hysterectomy had resulted in a loss of bladder musculature on the visceral surfaces of this structure. HIPEC with 80 mg of cisplatin and 24 mg of doxorubicin into the peritoneal space were delivered at 41.5–42.5 °C in the whole abdomen. Ifosfamide 2080 mg was given by continuous infusion over these 90 min. Mesna 416 mg was given 15 min prior to HIPEC and then 4 h and 8 h after completion of the ifosfamide infusion [3]. A permanent end-colostomy was constructed in the left lower quadrant. A permanent intraperitoneal port was positioned in the left epigastrium.

The patient received 500 mg of pemetrexed in 1 l of 1.5 % dextrose peritoneal dialysis solution once a month for 5 months. The intraperitoneal pemetrexed was supplemented with 75 mg/m^2^ of intravenous cisplatin. The patient was maintained on folic acid 1 mg/day and vitamin B12 1000 units every 2 months. The patient tolerated the 5 cycles of NIPEC chemotherapy well and her intraperitoneal port was removed without incident [7].

Patient has been followed with CT scans of chest, abdomen and pelvis on a six-monthly basis since the completion of her treatments. As of April 1, 2023, five years after her diagnosis and 4 years after her cytoreductive surgery, the patient remains free of disease, without disability, and eating a regular diet. No additional treatments were administered after the NIPEC treatments.

**Fig. 2:**
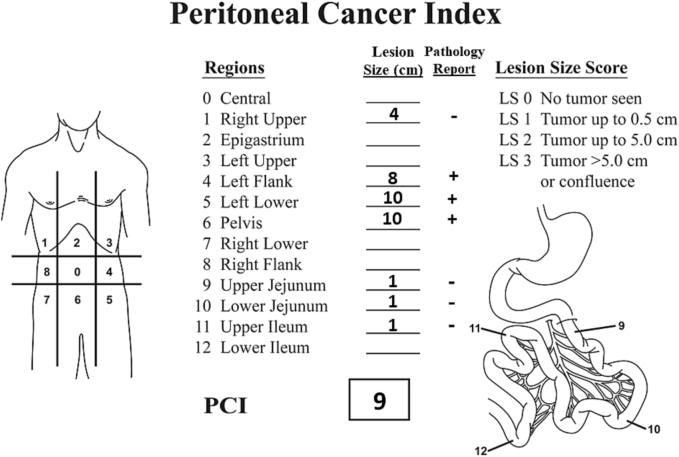
Peritoneal cancer index of the patient. Small nodules in abdominal-pelvic region 1 and attached to small bowel in regions 9, 10 and 11 showed fibrosis with calcification. Large partially necrotic masses in abdominal-pelvic regions 4, 5 and 6 showed sarcomatoid mesothelioma plus extensive calcification.

The pathology report from the cytoreductive surgery showed a heterogeneous response to the Nivolumab. A peritoneal cytology including a cell block was negative for mesothelioma. Two nodules from the small bowel showed “fibrotic tissue with calcification, negative for tumor.” One of these nodules was cystic. The mass from the right retrohepatic space in abdominopelvic region 1 showed a fibrotic nodule with focal calcification. No viable tumor cells were seen. A portion of the jejunum showed a fibrotic mass that was negative for metastatic tumor. All of the adhesions that were resected showed foreign body giant cell reaction with acute and chronic inflammation. No tumor was seen. The pelvic mass on the left side of the pelvis showed a high-grade malignant neoplasm compatible with a sarcomatoid peritoneal mesothelioma. The specimen from the pelvis included the complete pelvic peritoneum, rectosigmoid colon and rectum, apex of the vagina, and portion of the descending colon. A single nodule from the mass in the left paracolic sulcus measuring 1 cm in greatest diameter was positive by frozen section for malignant sarcomatoid type of mesothelioma. The pathology report was interpreted as a heterogeneous response. The small nodules were devoid of viable cancer cells with larger masses up to 10 cm in diameter showing viable sarcomatoid type of mesothelioma (Fig. 2).

## 4 Discussion

### 4.1 Combination of immunotherapy and CRS

In the management of high-grade malignancy with extensive peritoneal dissemination, the combination of systemic chemotherapy with a marked response plus complete cytoreductive surgery with HIPEC has been associated with a favorable outcome [1]. To our knowledge, this is the first report of a monoclonal antibody targeting the PD-1 receptor combined with cytoreductive surgery to achieve a long-term favorable outcome. A plausible explanation for this successful combination of treatments may be stated as follows: The immunotherapy was effective on smaller tumor nodules with an adequate blood supply which allows access for the PD-1 antagonist. Larger tumor nodules seem to have been partially treated in that they were partially necrotic and heavily calcified. However, disease progression was seen on CT and the patient's symptoms of bowel obstruction were increasing. There can be no doubt that the large pelvic masses and left-sided abdominal masses were progressing. The cytoreductive surgery combined with chemotherapy washing of the abdomen and pelvis combined with NIPEC long-term was able to eradicate this disease for a prolonged period of time. Currently, the patient is living a normal life with an end-colostomy without any evidence of disease.

Nivolumab is a human monoclonal antibody that targets the programmed death (PD-1) cluster of differentiation on the cell surface of suppressor T-cells (CTLA-4). Binding of PD-1 to its ligands on the T-cell surface downregulates the lymphocyte activity of the suppressor T-cell. Nivolumab inhibits the interaction of PD-1 to its ligands and thereby promotes immune responses and triggers antitumor activity. It has been used for a wide range of malignancies including malignant melanoma, non-small cell lung cancer, and gastric cancer with favorable responses.

### 4.2 Cisplatin can augment Nivolumab activity

Reports indicate that platinum drugs may enhance the effector immune response modulated by suppression of the PD-1 ligand. These encouraging results may extend the first line treatment of peritoneal mesothelioma with the hope of enhancing the antitumor response particularly in combination with current standard chemotherapy [8]. Currently, the standard of care for both thoracic and peritoneal mesothelioma is systemic cisplatin and systemic pemetrexed. [Anonymised] reported that the effects of long-term intraperitoneal pemetrexed with systemic cisplatin in a propensity matched trial is more successful than adjuvant treatments with both cisplatin and pemetrexed given systemically [9].

### 4.3 Immunotherapy before and HIPEC after CRS

The original strategy that supported treatment of peritoneal metastases advocated a combination treatment. The cytoreductive surgery using peritonectomy and visceral resections was to remove all visible evidence of disease. Only after complete CRS, the abdomen was flooded with a heated chemotherapy solution in hopes of eradicating residual disease. In the case reported here the combination treatment seems to be reversed. Initially, a systemic treatment with checkpoint inhibitors eradicated all of the small accumulations of sarcomatoid-predominant peritoneal mesothelioma. Then, the cytoreductive surgery removed large masses of tumor that presumably had inadequate blood supply to allow access of the immunotherapy treatments and progressed despite a partial response. The HIPEC and NIPEC were used in this highly motivated and fit patient in order to augment any additional anti-mesothelioma treatments required in order to eradicate this disease process. However histopathologically, the immunotherapy by eliminating all small components of the disease was, at least in large part, the treatment required to supplement the cytoreductive surgery.

### 4.4 Published activity of Nivolumab (anti-PD-1) for mesothelioma

To date, the results with PD-1 or PD-L1 inhibitors for mesothelioma have been modestly encouraging. In the MERIT study, MPM patients were treated with Nivolumab 240 mg IV every 2 weeks. The overall response rate was 29 % with a disease control rate of 67 %. In this study, a positive PD-L1 immunohistochemistry was associated with a higher progression-free survival of 7.2 months versus 2.9 months with a negative test. This trial led to regulatory approval in Japan for Nivolumab in pleural mesothelioma patients who progressed on prior chemotherapy [10]. In a study from the Netherlands, a single-armed phase II trial treated 34 patients with Nivolumab 3 mg/kg intravenously every 2 weeks. The 12-week disease control rate was 47 % with an overall response rate of 23.5 %. The median overall survival was 11.1 months. PD-L1 immunohistochemistry expression was not predictive for a response [11]. In the United Kingdom, 332 pleural mesothelioma patients who received first line platinum-based chemotherapy were randomized to Nivolumab versus placebo. The median overall survival was significantly higher with Nivolumab at 10.2 months versus 6.9 months in patients treated with cisplatin alone (HR 0.69; *p* = 0.0090) [12]. Some other immunotherapy agents have shown activity. Pembrolizumab showed an overall response rate of 20 % [13]. Avelumab (anti-PD-L1) showed a 9 % overall response rate with a 52-month median duration of response. Patients with PD-L1 immunohistochemistry greater than 5 % had a higher overall response rate of 19 % compared to an overall response rate of 7 % in PD-L1 receptor negative patients [11]. In conclusion, some prolongation of survival and an augmented response to cisplatin is expected in a low percentage of patients who receive PD-1 or PD-L1 antagonist treatment.

### 4.5 Immunotherapy benefit may depend on blood supply to a tumor mass

The review of the literature concerning PD-1 inhibitors or PD-L1 inhibitors for treatment of peritoneal mesothelioma is not impressive. Few responses are expected when Nivolumab is used as a single agent to treat this disease. What is important about this case report is the long-term benefit which can be achieved if a patient with strong radiologic evidence of response is then taken to the operating room for complete removal of residual tumor. The patient we presented showed stable disease in the small deposits of peritoneal metastases. She showed both radiologic evidence and symptoms of gross recurrence within the large tumor masses within the pelvis. This combination of systemic response, even if it was not complete, combined with a complete cytoreduction plus perioperative chemotherapy to mitigate against any tumor spillage that is likely to occur with the resection of such massive pelvic disease, is the combination of clinical findings which merits this aggressive approach. At least in this one patient, response to Nivolumab, despite progression of the large tumor masses, plus complete cytoreduction resulted in a 5-year disease-free survival. Prior to surgery the patient had weeks at most to live.

## 5 Conclusion

The combination of Nivolumab and CRS resulted in a long-term survival in this patient with sarcomatoid-predominant peritoneal mesothelioma. The possible role of cisplatin should also be mentioned as it may augment a release of PD-1 from inhibition. Access of the PD-1 antagonist to smaller nodules with adequate blood supply and its exclusion from large partially necrotic nodules was proposed. To my knowledge, this is the first successful report of a PD-1 agonist plus CRS resulting in long-term survival in a patient with high-grade peritoneal mesothelioma.

## Sources of support

Secretarial and administrative support from the Foundation for Applied Research in Gastrointestinal Oncology (FARGO).

## Ethical approval

[Anonymised] Health Institutional Review Board has determined that a case report of less than three [3] patients does not meet the DHHS definition of research (45 CFR 46.102(d)(pre-2018)/45 CFR46.102(l) (1/19/2017)) or the FDA definition of clinical investigation (21 CFR 46.102(c)) and therefore are not subject to IRB review requirements and do not require IRB approval.

## Funding

Data management and secretarial support provided by [Anonymised].

## CRediT authorship contribution statement

Paul H. Sugarbaker: study concept or design, data collection, data analysis or interpretation, writing the paper.

## Consent

Written informed consent was obtained from the patient for publication of this case report. A copy of the written consent is available for review by the Editor-in-Chief of this journal on request.

## Registration of research studies

This study was registered as a case report on the www.researchregistry.com website with UIN researchregistry8983.

## Guarantor

Paul H. Sugarbaker, MD.

## Provenance and peer review

Not commissioned, externally peer-reviewed.

## Declaration of competing interest

The author has no disclosures to declare.
